# Normative values of tear film parameters in brachycephalic dogs using OSA-Vet^®^: meniscometry and TBUTNI

**DOI:** 10.1007/s11259-026-11182-8

**Published:** 2026-03-27

**Authors:** Jéssica N. Voitena, Tatiane O. C. Marinho, Fabiano Montiani-Ferreira, Daniela N. Cremonini, João L. V. Chiurciu, Nadja S. Jesus, Ana P. V. B. Pohle, Olicies da Cunha, Fábio L. C. Brito

**Affiliations:** 1 Universidade Federal do Paraná (UFPR), Setor Palotina, Palotina, PR Brazil; 2 Faculdade Qualittas, São Paulo, SP Brazil; 3 Universidade Federal do Paraná (UFPR), Curitiba, PR Brazil; 4 Universidade São Judas Tadeu, São Paulo, SP Brazil

**Keywords:** Tear tests, Ocular surface analyzer

## Abstract

The precorneal tear film is critical for ocular surface health, and brachycephalic dogs are particularly predisposed to tear film instability due to their unique conformation. This study aimed to establish normative values for tear film parameters in healthy brachycephalic dogs using the OSA-Vet^®^, including Schirmer tear test-1 (STT-1), tear meniscus height (TMH), and non-invasive tear breakup time (TBUTNI). Forty brachycephalic dogs (66 eyes) with STT-1 values between 15 and 25 mm/min underwent complete ophthalmological examination and OSA-Vet^®^ evaluation. Data were analyzed for distribution, descriptive statistics, and correlations. Median values were 20 mm/min [19–22] for STT-1, 0.45 mm [0.36–0.62] for TMH, and 1.25 s [0.70–4.00] for TBUTNI. Spearman’s analysis revealed no significant association between STT-1 and TMH (ρ = 0.136, *p* = 0.277) or between TMH and TBUTNI (ρ = 0.046, *p* = 0.716). A weak positive correlation was observed between STT-1 and TBUTNI (ρ = 0.256, *p* = 0.038). These results demonstrate that even in dogs with preserved aqueous tear production, TBUTNI values remain low and variable, highlighting the predisposition of brachycephalic breeds to evaporative instability. TMH showed limited diagnostic value, underscoring the importance of multimodal tear film assessment. Previous studies have used OSA-Vet^®^ to evaluate ocular surface parameters across cephalic conformations. The present study focuses specifically on establishing clinically applicable reference values in phenotypically brachycephalic dogs screened for normal aqueous tear production.

## Introduction

The precorneal tear film plays a fundamental role in maintaining corneal transparency, ocular surface health, and visual quality in dogs, providing lubrication, antimicrobial protection, and metabolic support (Barabino et al. 2004). Quantitative and qualitative assessments of tear film parameters, such as tear volume, stability, and lipid layer quality, are essential for diagnosing and monitoring ocular surface disorders, including keratoconjunctivitis sicca (KCS) and evaporative dry eye (Iwashita et al. 2023). Recent advances in diagnostic technology have introduced objective and reproducible tools capable of evaluating these parameters in veterinary ophthalmology, including strip meniscometry, non-invasive tear breakup time (TBUTNI) and interferometry (Miyasaka et al. 2019; George and Mohan 2019).

Brachycephalic dog breeds are particularly predisposed to ocular surface disease due to conformational features such as shallow orbits, nasal folds, and lagophthalmos, which collectively define brachycephalic ocular syndrome (Plummer 2015; Costa et al. 2021). These anatomic abnormalities predispose to altered tear dynamics, meibomian gland dysfunction, and increased evaporative loss (Hisey et al. 2023). As a result, standardized reference values for tear film parameters are urgently needed for this population to enable early diagnosis and to guide therapeutic strategies (Packer et al. 2015; Bolzanni et al. 2020).

The OSA-Vet^®^ (Ocular Surface Analyzer, Veterinary Edition) is a non-invasive device adapted from human ophthalmology, designed to quantify tear meniscus height, TBUTNI and lipid layer interferometry in dogs (George and Mohan 2019). This system allows standardized, reproducible, and observer-independent measurements of tear film characteristics, facilitating both clinical diagnosis and research applications. The use of OSA-Vet^®^ has been validated in different cephalic conformations, showing promising results for both normative data collection and disease characterization (Voitena et al. 2024).

Despite growing awareness of brachycephalic ocular syndrome, there is a lack of normative reference data for advanced tear film diagnostic tools in these breeds. Most available studies are limited to Schirmer tear tests and phenol red thread methods, which do not provide sufficient information about tear stability or lipid layer quality (Miyasaka et al. 2019; Iwashita et al. 2023). The implementation of OSA-Vet^®^ offers an opportunity to establish standardized, reproducible values for meniscometry, TBUTNI and interferometry in brachycephalic dogs, bridging the gap between clinical need and diagnostic precision.

We hypothesized that clinically healthy brachycephalic dogs with preserved aqueous tear production (STT-1 within normal limits) would still exhibit reduced and highly variable tear film stability, as reflected by TBUTNI measurements, consistent with a predominately evaporative instability phenotype. We further hypothesized that TMH would show limited or no correlation with TBUTNI, indicating that surrogate metrics of tear quantity do not adequately represent tear film quality in this predisposed population.

This study provides clinically oriented normative OSA-Vet^®^ values for STT-1, TMH, and TBUTNI in a phenotypically defined cohort of brachycephalic dogs with preserved aqueous tear production, addressing a frequent diagnostic scenario and complementing previous publications such as Li et al. (2025).

## Materials and methods

### Ethics committee

The study was submitted and approved by the Animal Ethics Committee of the Federal University of Paraná - Sector Palotina (protocol n° 06/2021), according to the ethical principles of the Guidelines for Ethical Research in Veterinary Ophthalmology (GERVO). In addition, informed consent was obtained from all owners.

### Animals

A total of 40 brachycephalic dogs (23 females and 17 males), aged 1–9 years (mean ± SD: 5.0 ± 2.73 years), were enrolled. Based on STT-1 results, 66 eyes were included, as they exhibited values ranging from 15 to 25 mm/min. Inclusion criteria were established after a general clinical evaluation based on physical examination, medical history, and laboratory tests (complete blood count and liver and kidney function tests), in addition to a comprehensive ophthalmic examination including slit-lamp biomicroscopy, tonometry, and fundus examination. Brachycephalic dogs with no evidence of ocular and/or systemic diseases that could interfere with tear production and that were not receiving topical medications were included. Dogs presenting corneal ulcers, poor compliance with diagnostic procedures, or Schirmer tear test type 1 (STT-1) values greater than 25 mm/min or less than 15 mm/min were excluded.

All patients underwent a complete ophthalmological evaluated in the following order: OSA-Vet^®^ (TBUTNI, TMH), STT-1, and slit-lamp biomicroscopy. A 10-minute interval was maintained between tests to allow for tear film turnover (Sebbag et al. 2019). All ophthalmic examinations were performed by the same examiner (JNV) and performed on awake animals, in both eyes, with minimal physical restraint and without the use of medications. All examinations were performed under standardized conditions (temperature 18.5–25.5 °C; humidity ≈ 65%).

### Experimental design

#### Tear tests

The TBUTNI was assessed using the OSA-Vet^®^ by recording a 30-second video in which tear film disruption was identified through distortion of projected grid patterns. TMH was measured from three 15-second OSA-Vet^®^ videos per eye, selecting the best central meniscus image to calculate an average height in millimeters. STT-1 was performed using standardized filter paper placed in the lower lateral conjunctival sac for one minute, with tear production recorded in millimeters per minute using strips from a single batch to minimize variability (Voitena et al. 2024).

### Statistical analysis

Data distribution was assessed using the Shapiro–Wilk test and visual inspection of histograms. As all variables showed non-normal distributions, results are presented as median and interquartile range (IQR). To quantify the uncertainty around these descriptive reference values, 95% confidence intervals (CI) for the median were estimated using a non-parametric bootstrap approach (20,000 resamples).

To evaluate potential confounding and account for the inclusion of both eyes from the same subject, a linear mixed-effects model (LMM) was additionally fitted, with dog identity included as a random intercept and age and sex included as fixed effects. The mixed-effects analysis was used for confounder assessment only, as the primary objective of the study was to report descriptive reference values in a clinically homogeneous population. Statistical significance was set at *p* < 0.05.

## Results

A total of 66 eyes were evaluated, with no missing or duplicate data. The dog breeds included in the study were: Shih-Tzu (*n* = 22/40, 55%), Pug (*n* = 6/40, 15%), French Bulldog (*n* = 6/40, 15%), Lhasa Apso (*n* = 2/40, 5%), English Bulldog (*n* = 1/40, 2.5%), Chihuahua (*n* = 1/40, 2.5%), Boston terrier (*n* = 1/40, 2.5%) and Pekingese (*n* = 1/40, 2.5%).

**Table 1:** Descriptive statistics of tear film parameters (median [IQR])

Parameter	Median [IQR]	n	CI
STT-1 (mm/min)	20 [19–22]	66	20-21
TMH (mm)	0.45 [0.36–0.62]	66	0.40- 0.51
TBUTNI (s)	1.25 [0.70–4.00]	66	1.00-2.05

Abbreviations: Values presented as median [interquartile range]. STT-1 = Schirmer tear test-1; TMH = tear meniscus height measured by OSA-Vet; TBUTNI = non-invasive breakup time

**Fig. 1:**
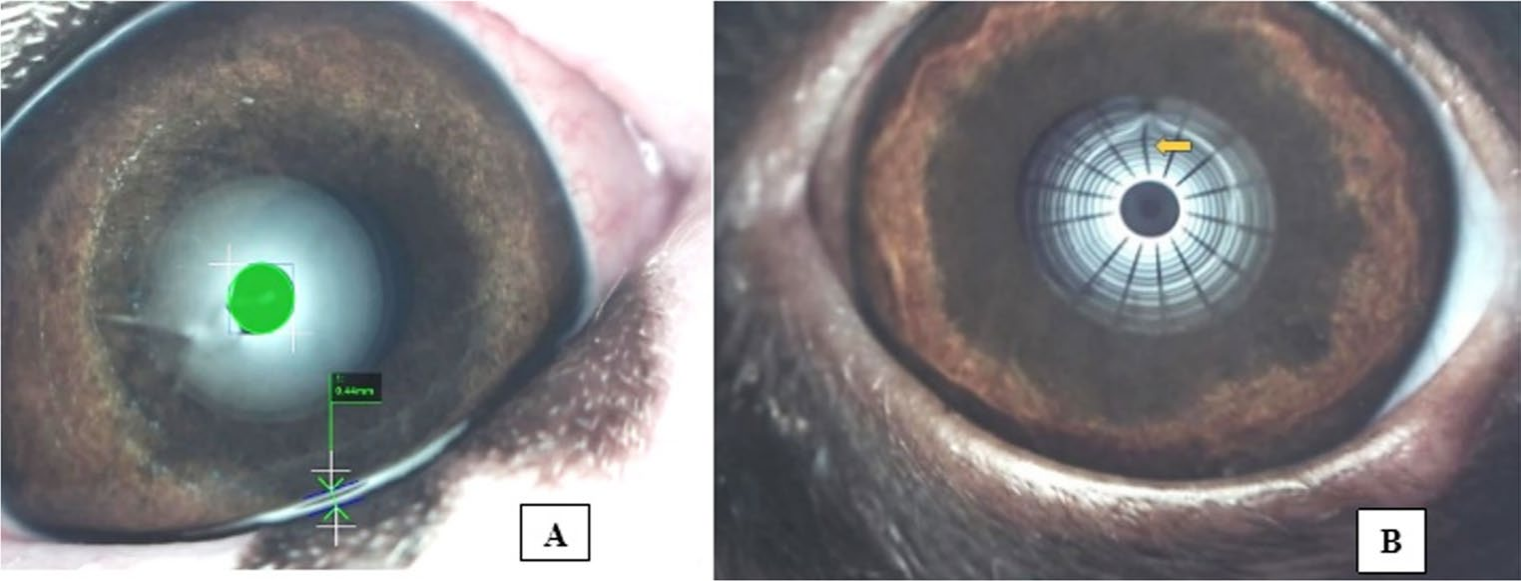
Image of the anterior segment, in a brachycephalic dog, obtained using the OSA-Vet^®^. **A** illustrates tear meniscus height (TMH) measurement, with the green-highlighted area indicating a height of 0.44 mm. **B** demonstrates the TBUTNI test, where grid distortion (yellow arrow) marks tear film irregularity and identifies the point of tear film breakup

Tear film parameters are summarized as median and IQR, with corresponding 95% confidence intervals for the median. STT-1 showed a median of 20 mm/min (IQR 19–22; 95% CI: 20–21). TMH presented a median of 0.45 mm (IQR 0.36–0.62; 95% CI: 0.40–0.51) (Fig. 1). TBUTNI showed a median of 1.25 s (IQR 0.70–4.00; 95% CI: 1.00–2.05) (Table 1).

Linear mixed-effects modeling demonstrated no significant effect of sex on STT-1, TMH, or TBUTNI (all *p* > 0.05). Age was not significantly associated with STT-1 or TMH; however, a significant negative association between age and TBUTNI was identified (β = −0.61 s/year; *p* = 0.002), indicating reduced tear film stability with increasing age.

**Fig. 2:**
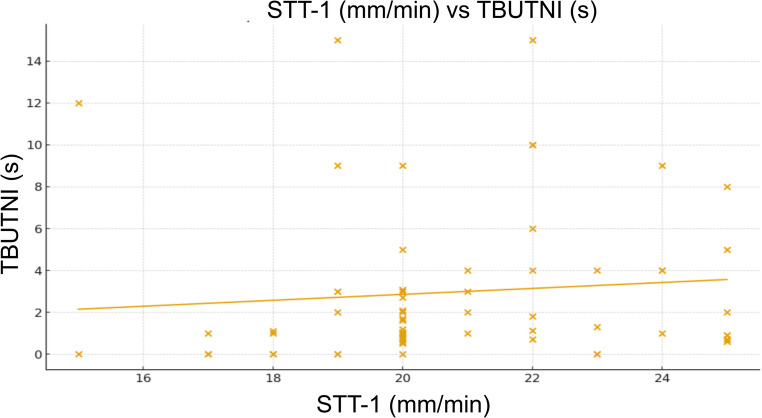
Scatterplot of Schirmer tear test-1 (STT-1, mm/min) versus non-invasive tear breakup time (TBUTNI, s) in brachycephalic dogs. Each point represents one eye. The regression line (orange) indicates a weak positive trend, suggesting minimal association between tear volume and tear film stability

Spearman’s correlation analysis showed no significant association between STT-1 and TMH (ρ = 0.136; *p* = 0.277; 95% CI: – 0.129 to 0.377). A weak positive correlation was observed between STT-1 and TBUTNI (ρ = 0.256; *p* = 0.038; 95% CI: − 0.006 to 0.501). The scatterplot illustrates (Fig. 2) the relationship between STT-1 and TBUTNI. TBUTNI values showed wide variability, ranging from near 0.5 to 15 s, while STT-1 values were mostly concentrated between 18 and 24 mm/min. No significant correlation was found between TMH and TBUTNI (ρ = 0.046; *p* = 0.716; 95% CI: − 0.222 to 0.308).

## Discussion

 The inclusion of bootstrap-derived 95% confidence intervals for the median provides an estimate of precision around the reported descriptive reference values without assuming normality. Mixed-effects modeling confirmed that sex does not act as a relevant confounder for any parameter. Although age was associated with reduced TBUTNI, this finding does not compromise the descriptive nature of the reference values and should be considered when interpreting tear film stability, particularly in older dogs

 The present study established reference values for OSA-Vet^®^ parameters in healthy brachycephalic dogs with STT-1 > 15 mm/min, reporting medians of 20 mm/min for STT-1, 0.45 mm for TMH, and 1.25 s for TBUTNI, with a weak but significant correlation between aqueous production (STT-1) and tear film stability (TBUTNI). This association suggests that even in dogs without quantitative tear deficiency, discrete variations in basal secretion may contribute to enhanced tear film stability, a biologically plausible finding considering the interaction between aqueous and lipid phases (Barabino et al. 2004; Li et al. 2025). The regression line showed a slight positive trend, indicating that higher STT-1 values were modestly associated with increased TBUTNI. However, the substantial dispersion and presence of outliers indicate a weak consistency in the relationship between the two variables. Thus, aqueous tear production (STT-1) did not reliably predict tear film stability (TBUTNI). From a clinical perspective, the result supports the concept that tear quantity does not necessarily reflect tear film stability, particularly in brachycephalic dogs, in which anatomical features and qualitative components of the tear film play a predominant role

 When compared with the literature, the STT-1 values obtained are consistent with previous reports describing mean values above 15 mm/min in normal dogs, including Beagles and Shih Tzus, supporting the cutoff adopted in this study (Kim et al. 2022; Silva et al. 2024). However, the TBUTNI values were lower and more variable than those reported in Beagles, in which normative values tend to be more stable and prolonged (Kim et al. 2022). This discrepancy likely reflects the peculiarities of brachycephalic conformation, such as larger palpebral fissures, increased ocular exposure, and less effective blinking (Costa et al. 2021; Palmer et al. 2021). In contrast, TMH showed no significant correlation with either STT-1 or TBUTNI, consistent with meniscometry studies demonstrating low reproducibility and weak association with aqueous tear secretion (Miyasaka et al. 2019)

 Mechanistically, these results reinforce that the tear film in brachycephalic dogs is more prone to evaporative instability even in the presence of preserved aqueous production (Sebbag and Sanchez 2022). The lipid layer, determined largely by meibomian gland function, plays a central role in maintaining TBUTNI, and subtle alterations in this interface may explain the variability observed (Hisey et al. 2023; Ng et al. 2024). Studies in dogs with meibomian gland dysfunction confirm that glandular dropout and lipid layer thinning are associated with reduced stability (Jeong et al. 2022), which is consistent with the finding of reduced and dispersed TBUTNI in this cohort. Moreover, the lower blink frequency in brachycephalic dogs may exacerbate tear film dispersion and ocular surface exposure, amplifying the clinical impact of borderline TBUTNI values (Williams and Denny 2020)

 From a clinical standpoint, the application of OSA-Vet^®^ in healthy brachycephalic dogs provides normative data useful for interpreting subtle tear film alterations, particularly in cases where STT-1 alone may not detect significant instability (Voitena et al. 2024; Li et al. 2025). Although TBUTNI values of 1–2 s fall within the normal range, they should be interpreted with caution in this group, as they may indicate a predisposition to ocular surface dysfunction when combined with clinical signs (Sebbag and Sanchez 2022). The lack of correlation between TMH and other parameters further highlights that isolated evaluation of tear meniscus height, while practical, should not be considered a robust predictor of stability or tear volume in dogs (Rajaei et al. 2018; Miyasaka et al. 2019)

 This study has several strengths, including the specific evaluation of healthy brachycephalic dogs and standardized application of OSA-Vet^®^, allowing for better understanding of tear film physiology in this population. Nonetheless, some limitations must be acknowledged. The inclusion criterion (STT-1 > 15 mm/min) restricted the analysis to dogs with preserved aqueous secretion, limiting extrapolation to hypolacrimic individuals (Bolzanni et al. 2020). Furthermore, the sample of clinically normal dogs may not reflect patients affected by brachycephalic ocular syndrome, in which instability is typically more severe (Sebbag and Sanchez 2022). Additionally, the intrinsic variability of TBUTNI and potential influence of environmental factors, such as relative humidity, could not be fully controlled (Yoon et al. 2020)

 Future research should focus on longitudinal studies correlating OSA-Vet^®^ parameters with clinical outcomes such as keratitis and corneal pigmentation in brachycephalic populations (Cirla et al. 2025). Multicentric validation with stratification by breed, age, and reproductive status may broaden the clinical applicability of reference values and identify more precise risk thresholds (Faghihi and Rajaei 2023). Moreover, integration of interferometry with complementary methods such as osmolarity and meibography may enhance the characterization of instability mechanisms across different conformational profiles (Ng et al. 2024; Hisey et al. 2023)

 Although both eyes were included for some dogs, a linear mixed-effects model incorporating dog identity as a random effect was applied to assess potential non-independence. Future studies with larger samples may allow age-stratified reference values

## Conclusion

 This study established reference values for STT-1, TMH, and TBUTNI in healthy brachycephalic dogs using the OSA-Vet^®^. The results showed low and variable tear film stability (TBUTNI) despite adequate aqueous tear production, indicating a predisposition to tear film evaporation in this population. TMH demonstrated limited correlation with the other parameters, reinforcing the importance of a multimodal assessment. The established values provide a clinically relevant standard for interpreting ocular surface stability, supporting early detection of subclinical dysfunction and serving as a basis for future studies and more precise diagnostic and therapeutic strategies

## Data Availability

No datasets were generated or analysed during the current study.
